# A Homozygous *NDUFS6* Variant Associated with Neuropathy and Optic Atrophy

**DOI:** 10.3233/JND-230181

**Published:** 2024-03-05

**Authors:** Andrea Gangfuß, Philipp Rating, Tomas Ferreira, Andreas Hentschel, Adela Della Marina, Heike Kölbel, Albert Sickmann, Angela Abicht, Florian Kraft, Tobias Ruck, Johann Böhm, Anne Schänzer, Ulrike Schara-Schmidt, Teresa M. Neuhann, Rita Horvath, Andreas Roos

**Affiliations:** a Department of Pediatric Neurology, Centre for Neuromuscular Disorders, Centre for Translational Neuro- and Behavioral Sciences, University Duisburg-Essen, Essen, Germany; b Department of Ophthalmology, University Duisburg-Essen, Essen, Germany; c Department of Clinical Neurosciences, John Van Geest Centre for Brain Repair, School of Clinical Medicine, University of Cambridge, Cambridge, UK; d Leibniz-Institut für Analytische Wissenschaften – ISAS – e.V. Dortmund, Germany; e Department of Neurology, Friedrich-Baur Institute, Munich, Germany; f MGZ - Medizinisch Genetisches Zentrum, Munich, Germany; g Institute of Human Genetics und Genomic Medicine, RWTH-Aachen University, Aachen, Germany; h Department of Neurology, Medical Faculty, Heinrich Heine University Düsseldorf, Düsseldorf, Germany; i IGBMC (Institut de Génétique et de Biologie Moléculaire et Cellulaire), Inserm U1258, CNRS UMR7104, Université de Strasbourg, Illkirch, France; j Institute of Neuropathology, Justus Liebig University, Giessen, Germany; k Children’s Hospital of Eastern Ontario Research Institute, Ottawa, ON, Canada

**Keywords:** Charcot-Marie-Tooth disease, axonal neuropathy, *NDUFS6*, white blood cell proteomics

## Abstract

**Background:**

The NADH dehydrogenase [ubiquinone] iron-sulfur protein 6 (*NDUFS6*) gene encodes for an accessory subunit of the mitochondrial membrane respiratory chain NADH dehydrogenase (complex I). Bi-allelic *NDUFS6* variants have been linked with a severe disorder mostly reported as a lethal infantile mitochondrial disease (LMID) or Leigh syndrome (LS).

**Objective:**

Here, we identified a homozygous variant (c.309 + 5 G > A) in *NDUFS6* in one male patient with axonal neuropathy accompanied by loss of small fibers in skin biopsy and further complicated by optic atrophy and borderline intellectual disability.

**Methods:**

To address the pathogenicity of the variant, biochemical studies (mtDNA copy number quantification, ELISA, Proteomic profiling) of patient-derived leukocytes were performed.

**Results:**

The analyses revealed loss of *NDUFS6* protein associated with a decrease of three further mitochondrial NADH dehydrogenase subunit/assembly proteins (NDUFA12, NDUFS4 and NDUFV1). Mitochondrial copy number is not altered in leukocytes and the mitochondrial biomarker GDF15 is not significantly changed in serum.

**Conclusions:**

Hence, our combined clinical and biochemical data strengthen the concept of *NDUFS6* being causative for a very rare form of axonal neuropathy associated with optic atrophy and borderline intellectual disability, and thus expand (i) the molecular genetic landscape of neuropathies and (ii) the clinical spectrum of *NDUFS6*-associated phenotypes.

## INTRODUCTION

Since 2004, 12 patients have been described with pathogenic variants in NADH ubiquinone oxidoreductase subunit S6 encoded by the *NDUFS6* gene [1–7]. Dysfunction of this complex I subunit of the respiratory chain is one cause of mitochondrial complex I deficiency (MCID) [8], leading to a severe disease mostly reported as a lethal infantile mitochondrial disease (LMID) or Leigh syndrome (LS) [1–5] with onset before 6 months of life and a progressive course [6]. The eight patients described by Kirby, Spiegel, Li and colleagues died aged less than 28 days due to central hypoventilation or ineffective treatment of severe lactic acidosis, respectively [1, 5, 7] (Supp. Tab. 1). Mitochondrial diseases often present with fatal infantile phenotypes but over the last years some authors reported isolated axonal neuropathy phenotypes, e.g. for *SCO2 [9]* and *MTATP6* [10].

## MATERIALS AND METHODS

DNA was extracted from peripheral blood (3.5 ml EDTA). Whole exome enrichment was performed with the Twist Human Comprehensive Exome Kit (Twist Biosciences). Massively parallel sequencing was carried out on a Novaseq 6000 system (Illumina, San Diego, CA) as 150 bp paired-end runs using v1.5 SBS chemistry. Exome enrichment-based SNV, INDEL and CNV calling was conducted using varvis® 1.19.3 (Limbus Medical Technologies GmbH, Rostock). Only SNVs and small INDELs in the coding and flanking intronic regions 106 (±50 bp) were evaluated. The analysis of the parental samples was performed equally by whole exome enrichment and the Twist Human Comprehensive Exome Kit (Twist Biosciences) (see above). The evaluation was focused and targeted for the *NDUFS6* variant. Pre-screened genes via panel analyses for Leber Hereditary Optic Neuropathy (LHON): *MT-ND1* (m.3460 G < A), MT-ND4 (m.11778 G > A), *MT-ND6* (m.14484T > C) and sequencing of *DNAJC30* (NM_032317) (hotspots). Gene panel for optic atrophy contained *ACO2, AFG3L2, C12ORF65, CISD2, DNM1* *L, DNAJC30, ELOVL1, FDXR, MCAT, MFN2, MIEF1, MTPAP, NBAS, NDUFS2, NR2F1, OPA1, OPA3, PRPS1, RTN4IP1, SLC25A46, SPG7, SSBP1, TIMM8A, TMEM126A, WFS1, YME1L1.*

A three-millimeter skin punch biopsy was performed 10 cm above the lateral malleolus and fixated in Zamboni fixative. Quantification of intraepidermal nerve fiber density (IENFD) were performed at 50μm sections with immunofluorescence staining with antibody against PGP9.5 to detect small intraepidermal nerve fiber as described previously [9, 11, 12].

Protein was extracted from white blood cells and analyzed by Western blotting. Samples were first lysed in RIPA buffer (Sigma-Aldrich) with cOmplet, Mini, EDTA-free Protease Inhibitor Cocktail (Roche). Twenty micrograms of protein was run per lane on NuPAGE 4–12% Bis-Tris Protein Gels (Invitrogen), transferred to a PVDF membrane and blocked with 5% milk (tris-buffered saline, skimmed milk powder, 0.1% Tween-20) for 1 h at room temperature. Primary antibodies were incubated in 5% milk overnight at 4°C, washed, and incubated with appropriate HRP secondary antibodies for 1 h at room temperature. Blots were developed with SuperSignal West Dura Extended Duration Substrate (Thermo Scientific). As primary antibody, we used anti-*NDUFS6*. Secondary antibody was goat anti-rat IgG (H + L) (ThermoFisher, 31470).

For mtDNA copy number quantification, DNA was obtained from white blood cells using the DNeasy Blood and Tissue Kit by Qiagen, and quantified using a nanodrop 2000 spectrophotometer (ThermoFisher). The samples were then diluted with nuclease-free water to achieve a final concentration of 2 ng/μL. The mtDNA copy number was quantified through a duplex TaqMan qPCR assay, amplifying both MT-ND1 (mitochondrial target) and B2M (nuclear target) using a CFX96trademark Real-Time PC Detection System (Bio-Rad). The sequences for the probes and primers used can be found in supplementary table 3. The relative mtDNA copy number was calculated by subtracting the B2M cycle threshold (Ct) from the MT-ND1 Ct (*Δ*Ct = CtB2M - Ct ND1) per sample. Sample mtDNA copy number calculated relative to a control *Δ*Ct.

In addition, we evaluated the established mitochondrial biomarker Growth Differentiation Factor 15 (GDF15) using enzyme-linked immunosorbent assay (ELISA). For the quantification of human GDF15 (R&D, DGD150), we followed the manufacturer’s instructions. Although GDF15 has not been previously associated with nuclear-encoded complex I respiratory chain complex defects, it has been implicated as a biomarker in peripheral neuropathies [13].

Proteomic profiling on whole protein extracts derived from our index patient as well as his healthy family members (serving as controls) was carried out in a data-independent-acquisition mode and subsequent data analyses were carried out as described previously [14].

## RESULTS

Here, we describe for the first time homozygosity for a known pathogenic *NUDFS6* variant associated with a novel phenotype consisting of axonal neuropathy, optic atrophy and borderline intellectual disability. Of note, previously reported mutations in *NDUFS6* were linked to severe lethal infantile phenotypes. Over the last years, some authors reported purely axonal neuropathy associated with less severe mutations in other “mitochondrial genes”, e.g. for *SCO2* [9] or *MTATP6* [10].

**Fig. 1:**
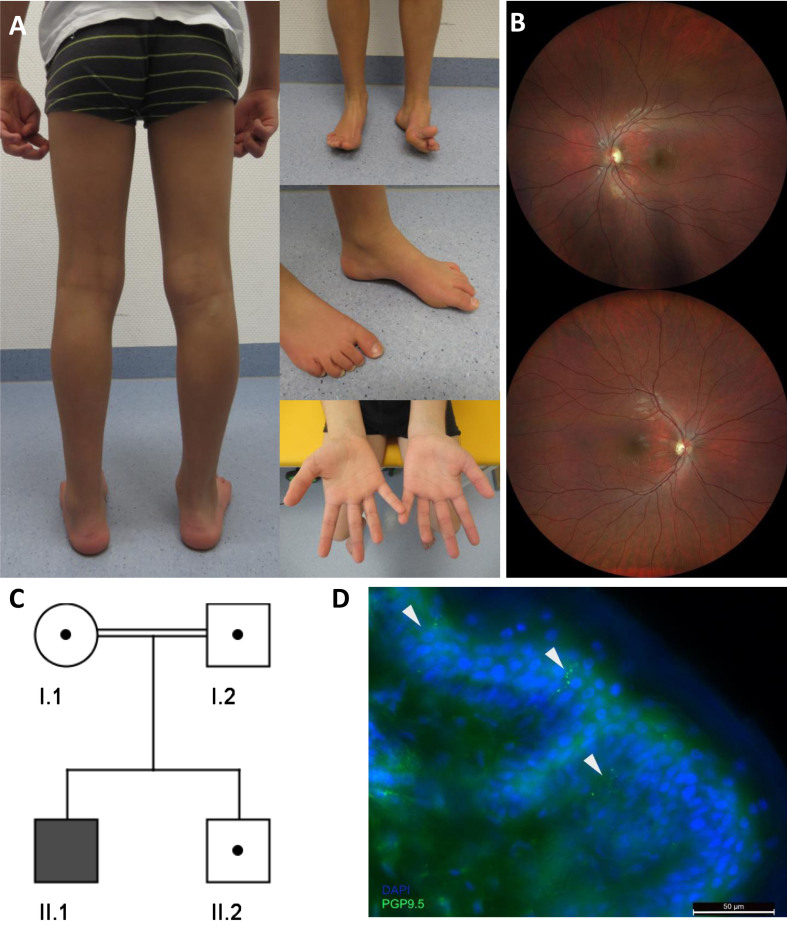
(A) Photographs of the index patient at the age of 9 years illustrate high arched feet (left > right) and reduced ability to lift both forefeet. (B) Ocular fundus showing bilateral partial optic atrophy (left > right). (C) Pedigree chart of the family described in this study. (D) Significant decrease of intraepidermal nerve fiber stain with PGP9.5 (arrows).

In the patient described here, the parents noticed an abnormal gait with frequent falls at the age of 7 years. Clinical examination at the age of 10 years showed pronounced distal muscle weakness of the lower limbs, high arched feet (left > right), and reduced ability to lift both forefeet (Fig. 1A) resulting in an abnormal gait with the inability to walk on the heels. In contrast, calve muscles showed no atrophy (Fig. 1A). Ankle jerk was absent, other deep tendon reflexes were normal. Vibration sensation was reduced (feet 4/8, hands 5/8) but coordination was normal. A standardised intelligence test (WISC-V) revealed borderline intellectual disability (IQ 70). Creatine kinase was normal, Blood lactate was mildly elevated (2,5 mmol/l [normal range 0,5–1,6]). Neurophysiologic studies showed results consistent with pronounced axonal sensory and motor neuropathy (reduced latency and amplitudes, mostly normal conduction velocities). Ophthalmologic examination (Fig. 1B) exhibited bilateral partial optic atrophy leading to an reduced visual acuity (left = 0.05 and right = 0.4) and altered visual evoked potentials (VEP, P100 amplitudes left smaller right, latency left 102 ms and right 105 ms). Cerebral Magnetic Resonance Imaging (MRI) was normal.

Supplementary table 1 presents a detailed clinical comparison of the patient described in this study and patients with *NDUFS6* variants previously described in the literature which highlights the difference in life expectancy in our patient [1–7].

Leber Hereditary Optic Neuropathy (LHON) was excluded by genetic analysis of the common mtDNA variants and a gene panel covering nuclear optic atrophy-associated genes was also normal (for analysed genes see above). *PMP22* deletion or duplication was excluded by MLPA. Whole exome sequencing identified a homozygous splice site mutation c.309 + 5 G > A in *NDUFS6* (NM_004553.6). The variant was assessed as “likely pathogenic” (ACMG class 4) and therefore compatible with the diagnosis “*NDUFS6*-associated mitochondrial diesease” in our patient. Next, segregation analysis of the healthy brother and both parents (cousins I°) was performed and revealed a heterozygous carrier status in all of them (Fig. 1C). Of note, Rouzier and colleagues described a patient showing compound heterozygosity for this variant in *NDUFS6* (c.309 + 5 G > A and c.343T > C) and functionally demonstrated the pathogenicity of the intronic c.309 + 5 G > A variant. Indeed, this variant strongly affects splicing, leads to exon 3 skipping, and results in a major decrease of the *NDUFS6* protein level [2]. Therefore, we suggest that the c.309 + 5 G > A variant is causative for the patient’s symptoms.

To further prove the pathogenicity of the homozygous *NDUFS6* variant identified in our index patient, we studied small nerve fibers and performed functional studies including determination of mitochondrial copy number in addition to proteomic studies on white blood cells and ELISA based quantification of GDF15 has been undertaken as described previously [13].

Quantitiave analysis of IENFD confirmed an involvement of small intraepidermal nerve fibers. The IENFD of 3.8 fibers/mm was significantly decreased in our patient compared to the published normative values for adults (6.1–10.9 fibers/mm) [11]. Moreover, IENFD is expected to be even higher in younger children [12]. Reduced density of small fibers thus indicates the presence of axonal sensorimotor neuropathy (Fig. 1D).

**Fig. 2:**
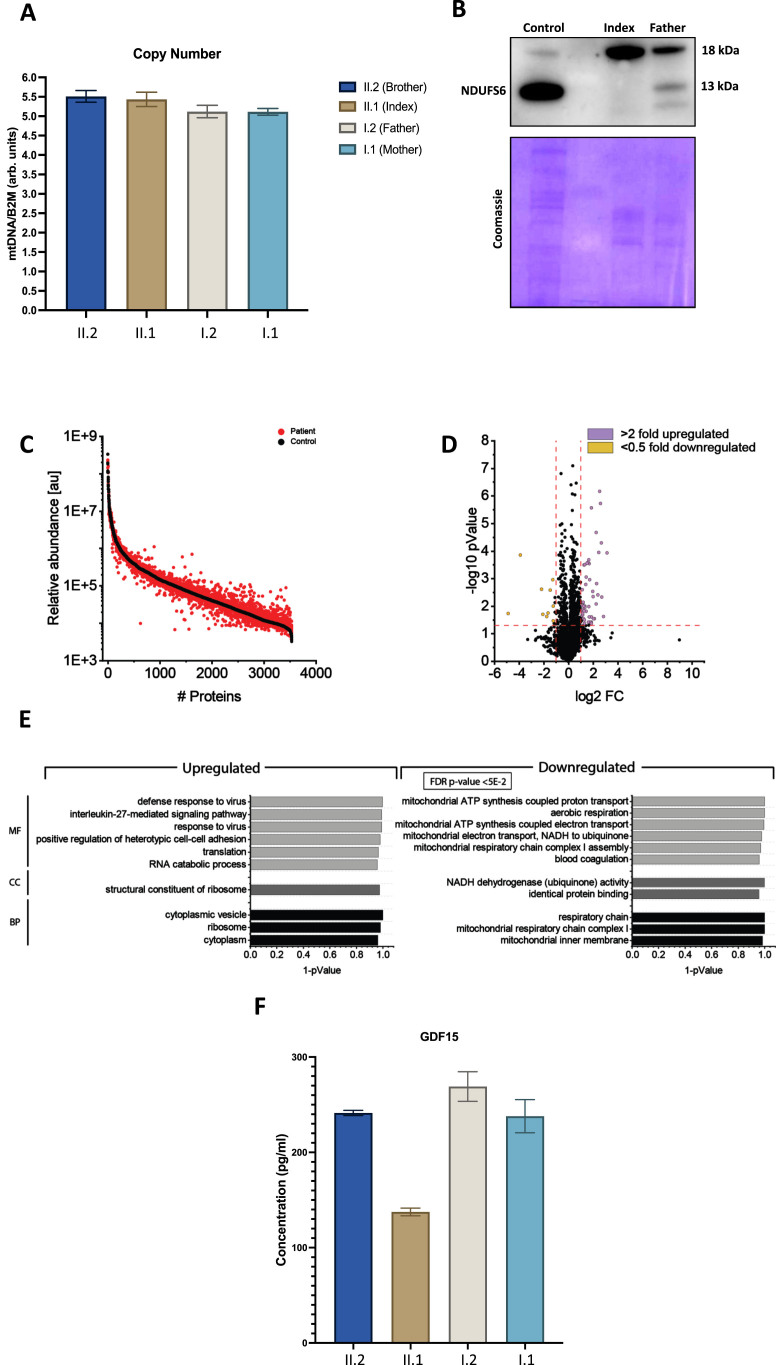
Molecular studies on white blood cells derived from our index patient and family members. (A) Quantitative PCR based determination of mitochondrial copy numbers did not reveal significant differences between the index and its family members. (B) Immunoblot studies of NUDFS6 revealed an absence of NDUFS6 (13 kDa) in protein extracts of white blood cells derived from the index, and decreased level of NDUFS6 in the heterozygous father compared to the level detected in control human fibroblasts. A smaller band on the Western blot may potentially represent an aberrantly spliced transcript. The 18 kDa band is unspecific. Coomassie staining was performed to demonstrate equal protein loading. (C) Abundance plot showing the dynamic range of all proteins identified in proteins extracts of white blood cells via liquid chromatography coupled to tandem mass spectrometry based on their relative quantification using always the 3 highest abundant peptides for each protein, allowing protein comparison within an experiment. All identified proteins of the controls (black) are sorted with decreasing abundance while the patient (red) was plotted in the same order to directly compare the different abundances. All identified proteins cover a dynamic range of eight orders of magnitude. (D) Volcano plot highlighting statistically significant increased proteins (purple dots) as well as decreased proteins (yellow dots). (E) Gene Ontology (GO) enrichment analyses for increased (left panel) and decreased (right panel) proteins separately. Decreased proteins clearly indicate a mitochondrial vulnerability upon reduced NDUFS6 level, as exemplified by our immunoblot studies. MF: molecular function of affected proteins, CC: cellular components affected by dysregulated proteins and BP: biological processes affected by dysregulated proteins.

Quantitative PCR based determination of mitochondrial copy number revealed no differences compared to the heterozygous healthy family members (Fig 2A). Immunoblot studies on whole protein extracts of white blood cells of the index patient showed absence of the NDUFS6 protein and it was also reduced in the heterozygous father. A faint smaller band may represent a mis-spliced NDUFS6 however we cannot fully confirm it (Fig. 2B).

Proteomic profiling holds the potential to identify pathophysiological processes in an unbiased manner by monitoring thousands of proteins in one experiment [14]. Recently, we successfully applied proteomic profiling on white blood cells to address the pathogenicity of a new *SCO2* variant associated with neuropathy and obtain new insights into the underlying pathophysiology [9]. After confirming the loss of expression of *NDUFS6* in white blood cells via immunoblotting (see above), we performed proteomic profiling on whole protein extracts derived from white blood cells of the index case in addition to the healthy brother and father (serving as controls) as described previously. Based on the applied data-independent approach, we quantified 3538 proteins (Fig 2C). Setting the boundaries for relevant protein increases and decreases to > 2.0 and < 0.5 fold of regular abundance, respectively, a total of 36 proteins (1.02% of the quantified proteins) showed a significant dysregulation: 10 proteins were decreased, whereas 26 were increased (Fig. 2D & Supp. Tab. 2). A Gene Ontology (GO)-term based in silico analysis (Fig. 2E) aimed to pinpoint pathophysiological processes upon presence of the homozygous *NDUFS6* variant. Notably, decreased proteins include three mitochondrial NADH dehydrogenase subunit/assembly proteins (NDUFA12, NDUFS4 and NDUFV1) indicative of altered mitochondrial respiratory chain complex I assembly (Fig. 2E), thus suggesting a pathophysiological effect of the homozygous *NDUFS6* variant. The neuropathy-related mitochondrial biomarker GDF15 did not show a significant change in our patient (Fig. 2F).

## DISCUSSION

Here, we describe a patient carrying a homozygous splice variant in *NDUFS6* leading to a milder phenotype than previously reported patients with *NDUFS6* variants: Our patient presented with a pronounced peripheral axonal neuropathy accompanied by involvement of small sensory nerve fibers, optic atrophy and borderline intellectual disability, however brain MRI was normal. Immunoblot studies revealed loss of the protein and proteomic findings further supported the pathogenicity of the identified splice-site variant by highlighting a concomitant decrease of three further mitochondrial NADH dehydrogenase subunit/assembly proteins (NDUFA12, NDUFS4 and NDUFV1), which are reflecting the dysfunction of the NDUFS6 protein.

Our data suggest that some cases present with a milder phenotype than these otherwise severe childhood onset mitochondrial diseases, possibly due to residual protein levels, however may be influenced by other genetic, epigenetic or environmental factors. Along this line, peripheral neuropathy as the leading symptom has already been reported in patients with mutations in some other primary mitochondrial disease genes (e.g. *SCO2*, *SURF1*) which are commonly associated with much more severe clinical presentations or even early death. An unbiased exome and genome sequencing approach is superior to targeted panel testing, as it enables the genetic diagnosis of these clinically unusual patients. However until this approach will become the standard diagnostic test we recommend that *NDUFS6* together with other mitochondrial genes (including *SCO2* and *SURF1*) should be included in panels for molecular genetic testing of neuropathies, intellectual disabillity and optic atrophy.

## Supplementary Material

Supplementary Table 1 Comparison of *NDUFS6* phenotypes. LIMD = lethal infantile mitochondrial disease; LS = Leigh syndrome; NT = not tested/performed; NS = not specified, LA = lactic acidosis; FTT = failure to thrive; PND = prenatal diagnosis, affected fetus terminated; M = male; F = female; NA = not applicable, yr = year, wk = week, mo = month, UNK = unknown

Supplementary Table 2 List of dysregulated proteins identified based on proteomic profiling including Uniprot accession number, fold of regulations, number of identified unique peptides and statistical significance of individual dysregulations.

Supplementary Table 3 Sequences of oligonucleotides and probes used for mtDNA copy number in patient samples.

## Data Availability

The data that support the findings of this study are available from the corresponding author upon reasonable request. Proteomic data have been uploaded to ProteomeXchange (identifier: PXD026283).
