# Supplementary material for: A Homozygous NDUFS6 Variant Associated with Neuropathy and Optic Atrophy
Source: J Neuromuscul Dis. 2024 Mar 5;11(2):485–91. doi: 10.3233/JND-230181 (PMC10977349; doi:10.3233/JND-230181)
Supplement: Supplementary Table 3 — Sequences of oligonucleotides and probes used for mtDNA copy number in patient samples. [file jnd-11-jnd230181-s003.docx]

| **Gene Target** | **Application** | **Forward primer (5’-3’)** | **Reverse primer (5’-3’)** |
| --- | --- | --- | --- |
| *MT-ND1* | mtDNA copy number | ACGCCATAAAACTCTTCACCAAAG | GGGTTCATAGTAGAAGAGCGATGG |
| *B2M* | mtDNA copy number | CACTGAAAAAGATGAGTATGCC | AACATTCCCTGACAATCCC |

| **Gene Target** | **Application** | **Fluorophore** | **Quencher** | **Probe Sequence** |
| --- | --- | --- | --- | --- |
| *MT-ND1* | mtDNA copy number | HEX | BHQ_1 | ACCCGCCACATCTACCATCACCCTC |
| *B2M* | mtDNA copy number | FAM | BHQ_1 | CCGTGTGAACCATGTGACTTTGTC |

Sequences of oligonucleotides and probes used for mtDNA copy number in patient samples.
